# Alginate-chitosan core-shell microcapsule cultures of hepatic cells in a small scale stirred bioreactor: impact of shear forces and microcapsule core composition

**DOI:** 10.1186/s13036-021-00265-6

**Published:** 2021-04-17

**Authors:** Shahla Khodabakhshaghdam, Ali Baradar Khoshfetrat, Reza Rahbarghazi

**Affiliations:** 1 Chemical Engineering Faculty, Sahand University of Technology, Tabriz, 51335-1996 Iran; 2 Stem Cell and Tissue Engineering Research Laboratory, Sahand University of Technology, Tabriz, 51335-1996 Iran; 3 Stem Cell Research Center, Tabriz University of Medical Sciences, Tabriz, Iran; 4 Department of Applied Cell Sciences, Faculty of Advanced Medical Sciences, Tabriz University of Medical Sciences, Tabriz, Iran

**Keywords:** Small scale stirred bioreactor, Hepatocytes, Mass production, Shear rate, Microencapsulation

## Abstract

A small scale stirred bioreactor was designed and the effect of different agitation rates (30, 60 and 100 rpm) was investigated on HepG2 cells cultured in alginate-chitosan (AC) core-shell microcapsule in terms of the cell proliferation and liver-specific function. The microencapsulated hepatic cells could proliferate well when they were cultured for 10 days at 30 rpm while the cell-laden microcapsules showed no cell proliferation at 100 rpm in the bioreactor system. Albumin production rate, as an important liver function, increased also 1.8- and 1.5- fold under stirring rate of 30 rpm compared to the static culture and 60 rpm of agitation, respectively. Moreover, In comparison with the static culture, about 1.5-fold increment in urea production was observed at 30 rpm. Similarly, the highest expressions of albumin and P450 genes were found at 30 rpm stirring rate, which were 4.9- and 19.2-fold of the static culture. Addition of collagen to the microcapsule core composition (ACol/C) could improve the cell proliferation and functionality at 60 rpm in comparison with the cell-laden microcapsules without collagen. The study demonstrated the hepatic cell-laden ACol/C microcapsule hydrogel cultured in the small scale stirred bioreactor at low mixing rate has a great potential for mass production of the hepatic cells while maintaining liver-specific functions.

## Introduction

Liver failure has been a significant problem for health care systems throughout the world [1]. Despite high regenerative potential of hepatocytes, whole liver transplants are still the only treatment option for patients with liver cirrhosis, liver cancer, or chronic liver disease [2, 3]. Unfortunately, lack of proper donor organs, cost of graft and the surgical complications strongly confine the number of patients that may benefit from liver transplants as majority of the individuals with liver failure in the world die because of long waiting for liver transplant [4]. Liver as a vital organ has a unique capacity for regeneration, and the regenerative potential of hepatocytes could be improved by using the appropriate biomaterial scaffolds for transplantation into the liver injured regions [5]. Hepatocytes are attachment-dependent cells and need an environment that mimics in vivo conditions [6]. Culture strategies which significantly promote cell–cell and cell–matrix contacts have been proved to enhance hepatic-specific functions [7]. Cell microencapsulation as a promising technology provides a 3D microenvironment, maintaining viability and functionality of cells by enabling the mutual exchange of nutrients and waste products. Moreover, some studies show alginate microcapsules with less than 500 μm diameter can provide adequate diffusion of oxygen to the microencapsulated cells [8–10]. Alginate microcapsules have also been used to confine cancer cells such as liver cancer cells and facilitate spontaneous formation of spheroids reproducing solid tumor properties [11]. Some studies have also attempted to engineer organoid models by microcapsules to recapitulate complex tumor microenvironments [12] as well as implantable therapeutics in tissue engineering [13]. Immune isolation of cells through microencapsulation has been investigated for many years, and microencapsulated organoid has been a long-sought solution for immune isolation of transplanted organoid [14]. Alginate-polycation microcapsules have been recently used for the immune protection of transplanted hepatocytes as well as hepatic tissue formation [15–18].

To obtain a 3D functional tissue-like graft, static cultivation is not applicable due to the limitations in cell density, nutrition and oxygen support. Bioreactors develop a dynamic cultivation system within a controlled environment providing the physicochemical requirements of a mass production of stem cells [5]. Many considerations, however, should be taken into account when stem cells are cultured in dynamic systems due to the lack of cell walls [19]. Culturing cell-laden 3D scaffolds within bioreactors can protect the cells from environmental shear forces in dynamic systems and may more accurately simulate the growth environment of stem cells in the human body [20, 21].

For cell therapy applications and in the case of hepatocytes culture, several bioreactors have been developed. Fluidized bed bioreactors are of the prevalent bioreactors which have recently been developed for hepatocyte culture. Lu, Zhang [22] incorporated fluidized bed bioreactors with alginate/chitosan microcapsules to provide a favorable 3D microenvironment for cell survival and functionality of hepatocytes. Despite the advantages of fluidized bed bioreactors for bio artificial liver system, the bioreactors have some shortcomings such as high perfusion rate which can disrupt microcapsules and even cause cell damage.

Stirred tank bioreactor has attracted much interest in stem cell proliferation due to its unique characteristics such as simplicity of running, different modes of operation, ability to exact control of the parameters such as pH, dissolved oxygen and metabolites and better mass transfer capability. In a stirred tank bioreactor, the mixing invoked by the stirrer improves the mass transfer and homogenizes bulk media conditions that can enhance cell growth [23]. Recent studies have shown that the dynamic culture of hepatocyte aggregates microencapsulated in high-viscosity alginate hydrogel in stirred bioreactors can develop an easily accessible 3D organization for long-term hepatic cultivation [24, 25]. Hydrodynamic shear stress, however, can negatively affect fate of cells and alter their morphologies and functionalities [7].

In our previous study, the alginate-based microcapsules coated with chitosan (AC) revealed a potential 3D platform for mass production of hepatic cells and collagen could improve the functional activity of hepatic cells inside the microcapsules [18]. To examine the effect of shear forces on the proliferation and functionality of microencapsulated hepatocytes in vitro, in this work, HepG2 cells were encapsulated in the AC microcapsules and then cultured under different stirring conditions in a small scale stirred bioreactor. Mechanical characteristic quantities such as micro-shear rate and impact of microcapsules collisions with each other and impeller were evaluated. Eventually, to examine the effect of the core matrix compositions on shear rate impact, the microencapsulated hepatocytes in the alginate-collagen hydrogels coated with chitosan were also cultured at 60 rpm agitation in the stirred bioreactor. Hepatic functional activities were evaluated by measuring albumin and urea secretion and gene expression of albumin and p450.

## Materials and methods

### Materials

Sodium alginate (medium viscosity, viscosity > 2000 cps for 2% aqueous solution at 25 °C), chitosan (medium molecular weight), calcium chloride, citrate sodium and HEPES were purchased from Sigma-Aldrich. High glucose-Dulbecco’s Modified Eagle’s Medium (H-DMEM), penicillin/streptomycin (pen/strep), fetal bovine serum (FBS), Phosphate-Buffered Saline (PBS) and trypsin-EDTA 0.25% were obtained from Gibco (France). Collagen type I was provided from SBPE Company (Iran).

### Cell microencapsulation and culture

To prepare cell-laden microcapsules, at first, the detached HepG2 cells (Pasture Institute, Iran) were suspended at a density of 2 × 10^6^ cells per ml of the hydrogel solution. Briefly, the alginate solution (1% w/v) containing cells was transferred into a 5 ml syringe and extruded through a 26 G flat-cut needle to 100 mM CaCl_2_ solution as cross linking bath. The microcapsules with average size of 500 ± 50 μm were prepared by applying 8 kV voltage using electrostatic extrusion method. The alginate-collagen (ACol) solution was prepared by mixing pre-cooled collagen (type I) solution with alginate solution having final concentrations of alginate 1% and collagen 0.125%, respectively. Then, the microcapsules were washed twice with CF-KRH solution and then suspended in 0.3% (wt.) chitosan solution (pH = 6.5–6.6) for 5 min to obtain chitosan coated microcapsules. Eventually, after washing with CF-KRH solution the microcapsules were cultured in high glucose-DMEM composed of 10% FBS and 1% antibiotics at 37 °C in 5% CO_2_ atmosphere.

HepG2 cells were successfully entrapped within the microcapsules without any visible loss. Each microcapsule had approximately 100–130 cells that were homogeneously dispersed within the microcapsule. The phase-contrast images showed that the cells tended to escape from the uncoated capsules within a few days. During the culture period, no cell leakage was observed and chitosan coating could provide a good stability for microcapsules.

### Mechanical properties of microcapsule

Mechanical property of the microcapsules was determined by a shear force test according to the method described by Tian, Han [26]. Briefly, about 100 microcapsules from AC and ACol/C hydrogels were prepared and then located into a 6-well plate before immersing in 3 ml of PBS. The microcapsules were agitated at the frequency of 240 rpm at 37 °C on the orbital shaker. The percentage of the ruptured microcapsules at various time points was counted with a phase contrast microscope (IX71, Olympus, Japan).

### Bioreactor

**Fig. 1:**
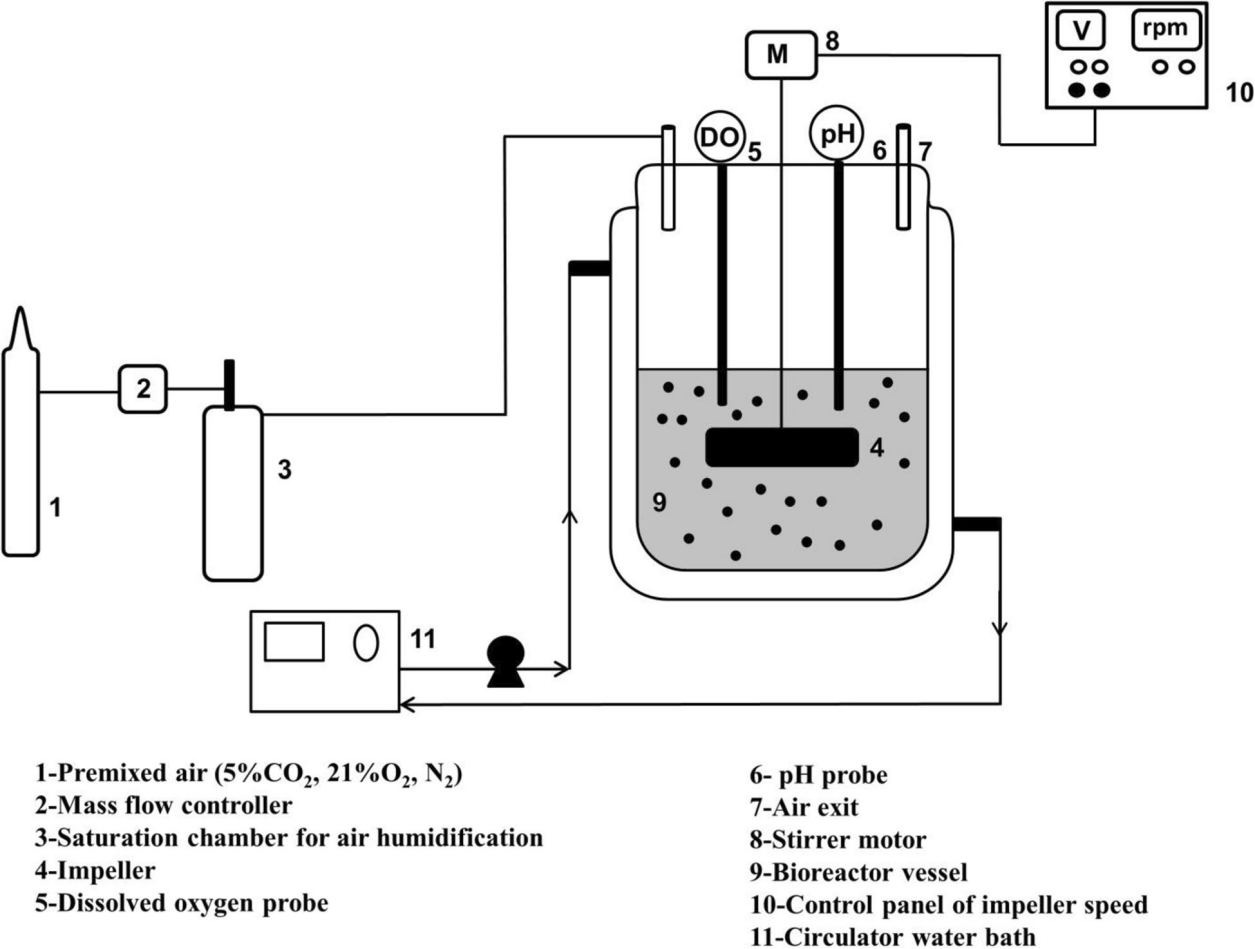
Schematic of bioreactor system

For bioreactor culture, a sealed cylindrical vessel in 90 ml working volume was equipped with flat blade paddle impeller rotated by an external motor (Fig. 1). The aspect ratio, the medium height (H) to the diameter of bioreactor (D), was selected 0.56. The diameter of impeller (d) was 3.5 cm, resulting in the ratio of the diameter of the stirrer’s blade to the diameter of the vessel (d/D) of 0.58. The culture medium was aerated with a 5% CO_2_/air mixture at a constant total gas flow of 0.1vvm. For elimination of bubble shears, the surface aeration was selected as the aeration system. Temperature was maintained at 37 °C by circulating water through the vessel jacket controlled by a water circulator having temperature controller (UC5000, Sahand Azar Co., Iran).

In brief, AC microcapsules after preparation were cultured 2 days in static culture and then suspended in the bioreactor with 5% loading. The 50% of the culture medium was replaced with fresh medium on days 1, 3, 6, 8 and 10, and some culture medium was stored in − 20 °C for analysis. Medium exchange strategy as replacing half of the culture medium at certain time intervals and CO_2_ as a part of gas flow could control pH at 7.5 ± 0.2. The agitation rates of 30, 60, 100 rpm were tested and compared with the static culture.

### Shear stress calculations

Agitation is essential for suspending cells or cell-laden scaffolds and achieving the homogeneity of nutrition and oxygen in bioreactors. It has been reported that sever agitation could be harmful for animal cells and cause cell death and dedifferentiation [27]. Based on the previous studies, the maximum shear stress imposed on a cell/aggregate could be estimated by the following equation [27–30]:

1 $$ {\tau}_{max}=5.33\rho {\left(\varepsilon \vartheta \right)}^{\frac{1}{2}} $$

The above equation can be used for the calculation of shear on microcapsules, where *ρ* is the medium density (kg/m^3^), *υ* is the kinematic viscosity of the medium (m^2^/s) and *ε* is the power dissipated per unit mass (W/kg) which is calculated by Eq. 2:

2 $$ \varepsilon =\frac{P}{V_{\mathrm{L}}.\rho } $$

where *P* is the power consumed (W) and *V*_L_ is the bioreactor working volume (m^3^). The consumed power can be obtained from Eq. 3 [31]:

3 $$ P={N}_p{N}^3{d}^5\rho $$

where *N* is the impeller speed (rps) and *d* is the impeller diameter (m). The value of *N*_*P*_ (the dimensionless power number) which is a weak function of impeller geometry and Reynolds number (*Re* > 1000), can be calculated from the Nagata correlation [31].

Shear depends on the geometric characteristics of the vessel and the impeller. The concept of an integrated shear factor (*ISF*) was developed by Sinskey, Fleischaker [32] to characterize shear field between the impeller and the vessel walls for mammalian cells in small scale stirred bioreactors (Eq. 4) [30, 33]:

4 $$ ISF=\frac{2\pi Nd}{D-d} $$

where *D* and *d* are the diameters of the impeller and the vessel, respectively.

### MTT assay for cell viability

Proliferation behaviors of HepG2 cells cultured in microcapsules were assessed in terms of mitochondria activity using MTT assay (Sigma–Aldrich), as described elsewhere [18]. Briefly, after being cultured for a predetermined period, the cells encapsulated in microcapsules were released by using a buffer solution containing 200 mM sodium bicarbonate and 60 mM sodium citrate. The released cells were collected by centrifugation at 1000 rpm for 5 min, and 1 ml MTT solution (5 mg/10 mL of medium) was then added before incubating the cells at 37 °C in a humidified atmosphere (95% air and 5% CO_2_) for 4 h. Afterward, the MTT solution was removed and replaced with 500 μL dimethyl sulfoxide (DMSO). Finally, the absorbance of the samples was measured at 570 nm using a spectrophotometer (CE2501, Japan). The absorbance values were normalized by number of microcapsules per samples. At least three samples were used for each group.

### Measurement of glucose consumption and lactate production

Glucose and lactate concentrations were measured by using kits and the automated analyzer (Cobas 6000, Germany) according to the manufacturer’s recommendations. The measurements were repeated three times. The amounts of lactate and glucose were measured in the supernatant of the culture medium taken from the bioreactor on days 1, 3, 6, 8 and 10 of culture.

### Urea and albumin synthesis tests

The secretion of albumin from hepatocytes was measured by a sandwich enzyme-linked immunosorbent assay (ELISA) using a human albumin ELISA kit (Padtan Elm, Iran) according to the manufacturer’s protocol. The optical density (OD) values were read at 450 nm using a microplate reader. The amount of albumin was calculated based on a standard curve plotted using a reference human albumin. The urea synthesis rate was determined by using the commercial urea UV kit (Pars Azmon, Iran) based on the Urease-GLDH method according to the manufacturer instructions. At least three samples were used for each group. The results were expressed as mg/dl at the indicated time points.

### Real-time polymerase chain reaction (PCR) analysis

Total RNA was extracted from the cells expanded from each group at the end of 10 days by using a RNA extraction kit (Cat no: YT9065, Yekta Tajhiz Azma). The integrity and level of isolated RNAs were determined by a Spectrophotometer (PicoPET01, Picodrop; UK), and then reverse-transcribed into cDNA by using cDNA synthesis kit (Cat no: YT4500). Primers for Albumin, GAPDH, P450 genes were designed by Oligo primer analysis software (Version 7.0) (Table 1). The transcription of genes was evaluated using SYBR Green Master Mix and Mic RealTime PCR System. This analysis was implemented in triplicates.

**Table 1:** The list of primers used for the gene expression analysis

Gene	Forward primer	Reverse primer
Albumin	TCAAGTGTGCCAGTCTCCAAA	GTCATCAGCACATTCAAGCAGA
P450	CTTCATCTACTGGCTCACCCC	TCTTCCGCACCTTCTCATCCT
GAPDH	AGCCAAAAGGGTCATCATCTCT	AGTCCTTCCACGATACCAAAGT

### Statistical analyses

Statistical analyses were conducted between independent groups using unpaired, two-tailed Student’s t-tests. The results were considered statistically significant at *p* < 0.05. Each data point represented in this study was the mean of three replicates.

## Results

### Influence of mixing rate and core composition on microencapsulated HepG2 cell proliferation

**Fig. 2:**
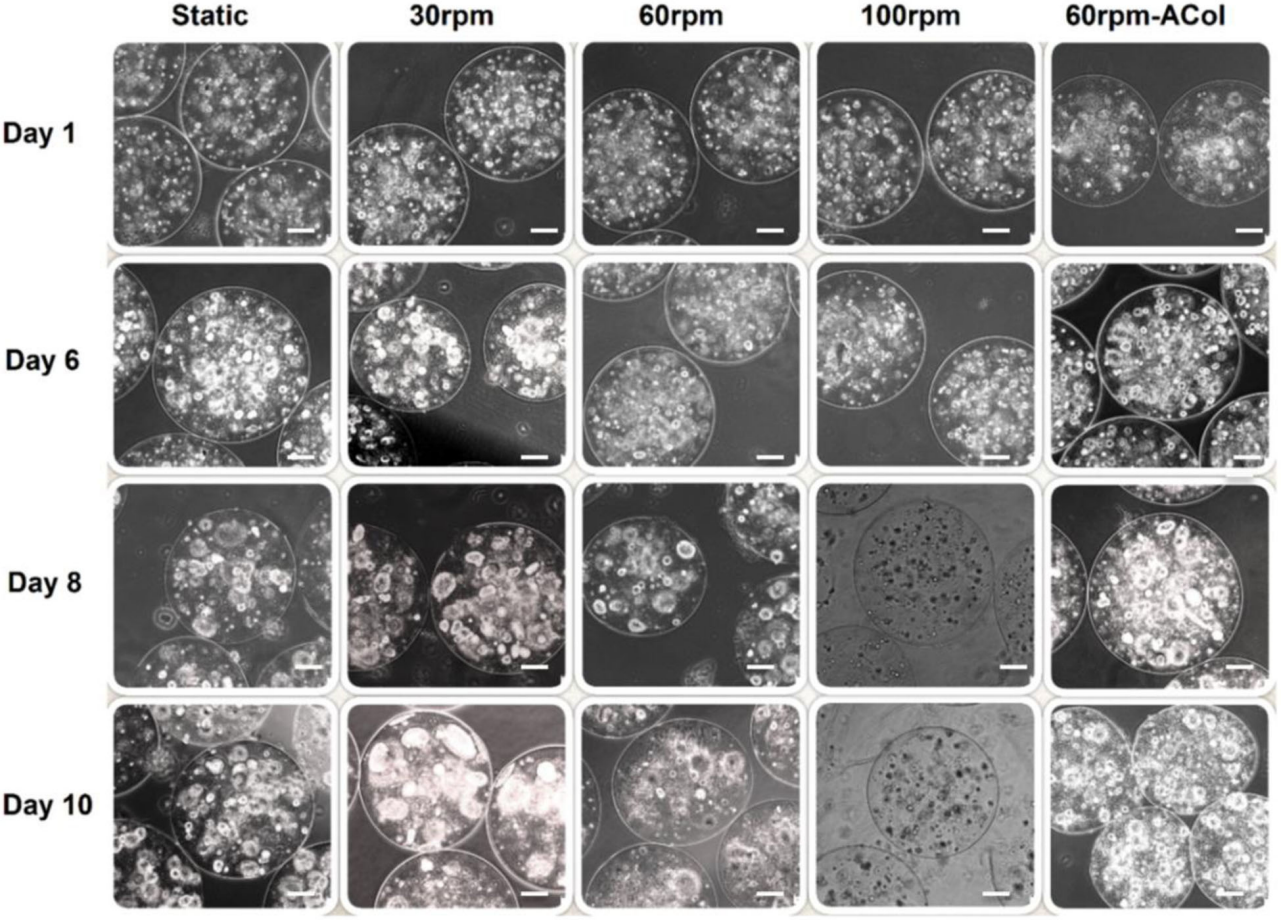
Phase-contrast microscopy images of microencapsulated HepG2 cells at initial cell density of 2 × 10^6^ cells/ml for microcapsules during 10 days culture period in the stirred bioreactor, (Scale bar: 100 μm)

**Fig. 3:**
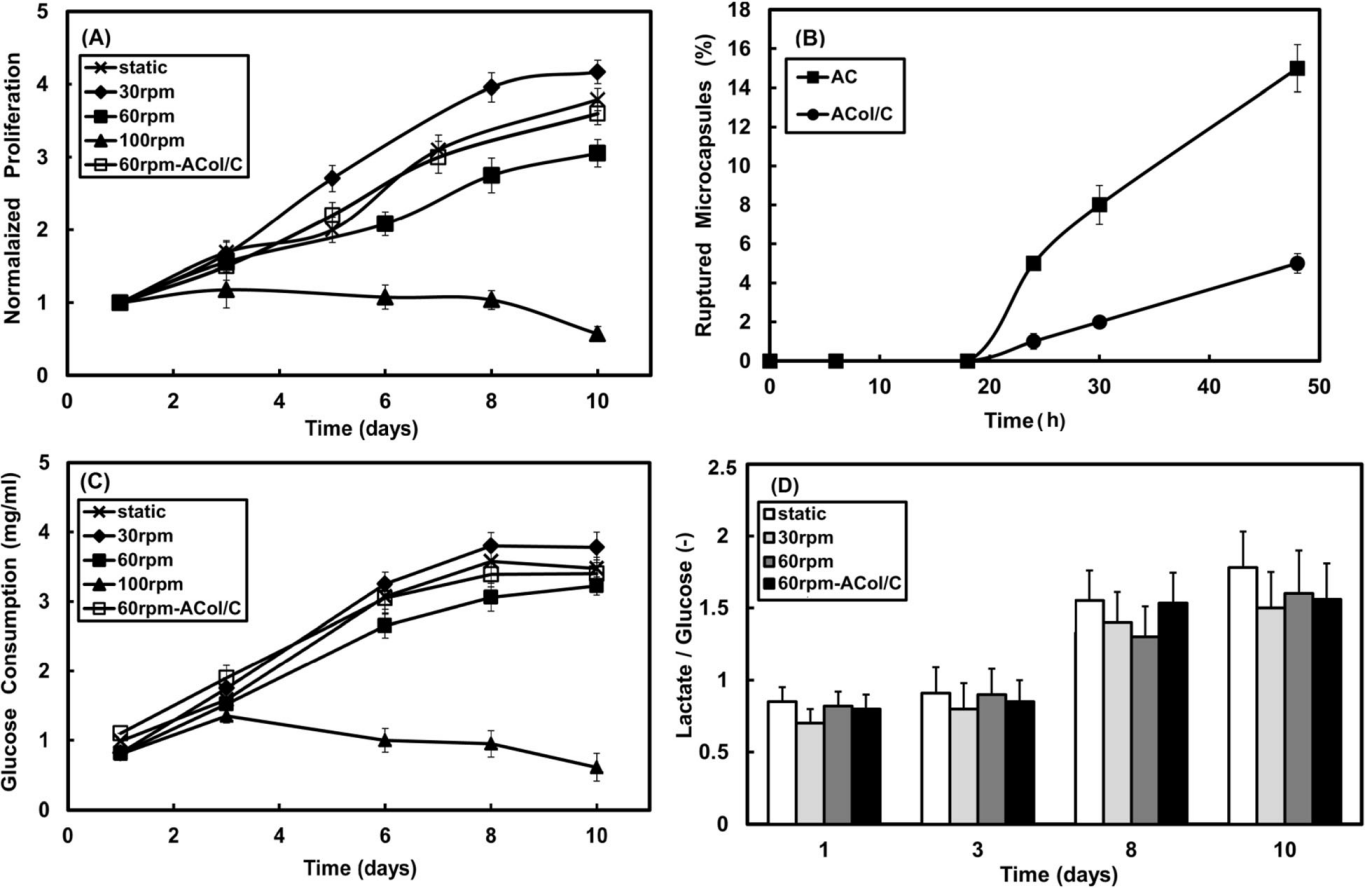
Proliferation of HepG2 cells in the microcapsules during 10 day culture in the stirred bioreactor (**a**), mechanical property of AC and ACol/C microcapsules (**b**), glucose consumption at the determined days before 50% exchanging of culture medium (**c**) and the ratio of lactate production to glucose consumption at determined days (**d**)

Changing mixing rate affected considerably the proliferation behaviors of HepG2 cells cultured in alginate-chitosan core-shell microcapsules in the small-scale stirred bioreactor system, and the size of cell aggregations was quite different at various mixing rates, as shown in Fig. 2. According to the results of MTT assays (Fig. 3a), the metabolic activity of microencapsulated HepG2 cells increased significantly at 30 rpm agitation speed during the cultivation period. As shown in Fig. 3a, the number of active cells increased during 10 days cultivation at the mixing rates of 30 and 60 rpm as well as static culture, whereas at 100 rpm speed after 4 days, no cell proliferation was observed. After 4 days, interestingly, significant changes in the microencapsulated cell activity were observed at 30 rpm agitation in comparison with the static and 60 rpm agitation cultures (*p* < 0.05). Cell expansion fold at the 30 rpm speed and static cultures were, respectively, 4.2- and 3.8-fold after 10 days of cultivation, indicating dynamic culture in low rates could develop a sufficient nutrient and oxygen mass transfers for the microencapsulated hepatic cells.

It is also evident from the results that existence of collagen in microcapsule core accelerated 20% cell proliferation after 10 days at 60 rpm agitation (*p* < 0.05), indicating the positive effect of collagen on the microencapsulated cell proliferation in dynamic culture.

Mechanical stability of the hydrogel microcapsules is an important parameter for cell culture in bioreactors under shear stress. Alginate is an anionic linear polysaccharide which can form hydrogels in the presence of some multivalent metal ions such as calcium ion. Chitosan as cationic polysaccharide can diffuse into three-dimensional alginate gel network. Under electrostatic interaction between protonated amino groups of chitosan and carboxyl groups of alginate, polyelectrolyte complex microcapsule membrane is formed. This membrane has been exhibited to play an important role in controlling the properties of AC microcapsules [34]. Figure 3b shows the mechanical stability of AC and ACol/C microcapsules. The percent of broken microcapsules was used as an indication of the mechanical stability of the microcapsules. After 48 h of shaking, the rupture percentage of microcapsules was observed 15 and 5% for the AC and ACol/C samples, respectively, revealing collagen improves the mechanical strength of AC microcapsule.

Phase-contrast microscopy images from the microcapsules collected from each condition coincided also with the results of the MTT assays. In fact, in addition to provide adhesion groups, collagen can protect the microencapsulated cells under higher shear forces in the dynamic culture.

### HepG2 cell glucose consumption and lactate production

As shown in Fig. 3c, based on the data up to day 3 it can be observed that glucose consumption of the cell-laden microcapsules showed no significant differences when they were cultured at 30 and 60 rpm except 100 rpm speed. After three days of dynamic culture, the hydrodynamic condition affected HepG2 cells and the glucose consumption was different. For example, glucose consumption at 30 rpm speed was approximately 11% higher than 60 rpm on day 3 (*p* < 0.05). After 3 days, the amount of glucose consumption at 30 rpm speed enhanced, indicating a proper condition for cells growth. The consumption of glucose at 100 rpm speed decreased after 3 days, which was in consistent with the results of the viability test (Fig. 3a).

The ratio of lactate production to glucose consumption is a better measure of cellular metabolism (Fig. 3d). Under hypoxic conditions, a single mole of glucose is broken down into two moles of lactate if enough oxygen is supplied to the cells and the ratio of lactate production to glucose consumption becomes less than two [35]. It was observed that the ratios were similar for all the cases up to day 3 and the ratios for all the cultures were around 0.9. In the late days of hepatocyte cultures, however, the static culture had the highest ratio indicating lower oxygen provided for the microencapsulated cells.

### Hepatic functions of microencapsulated HepG2 cells

**Fig. 4:**
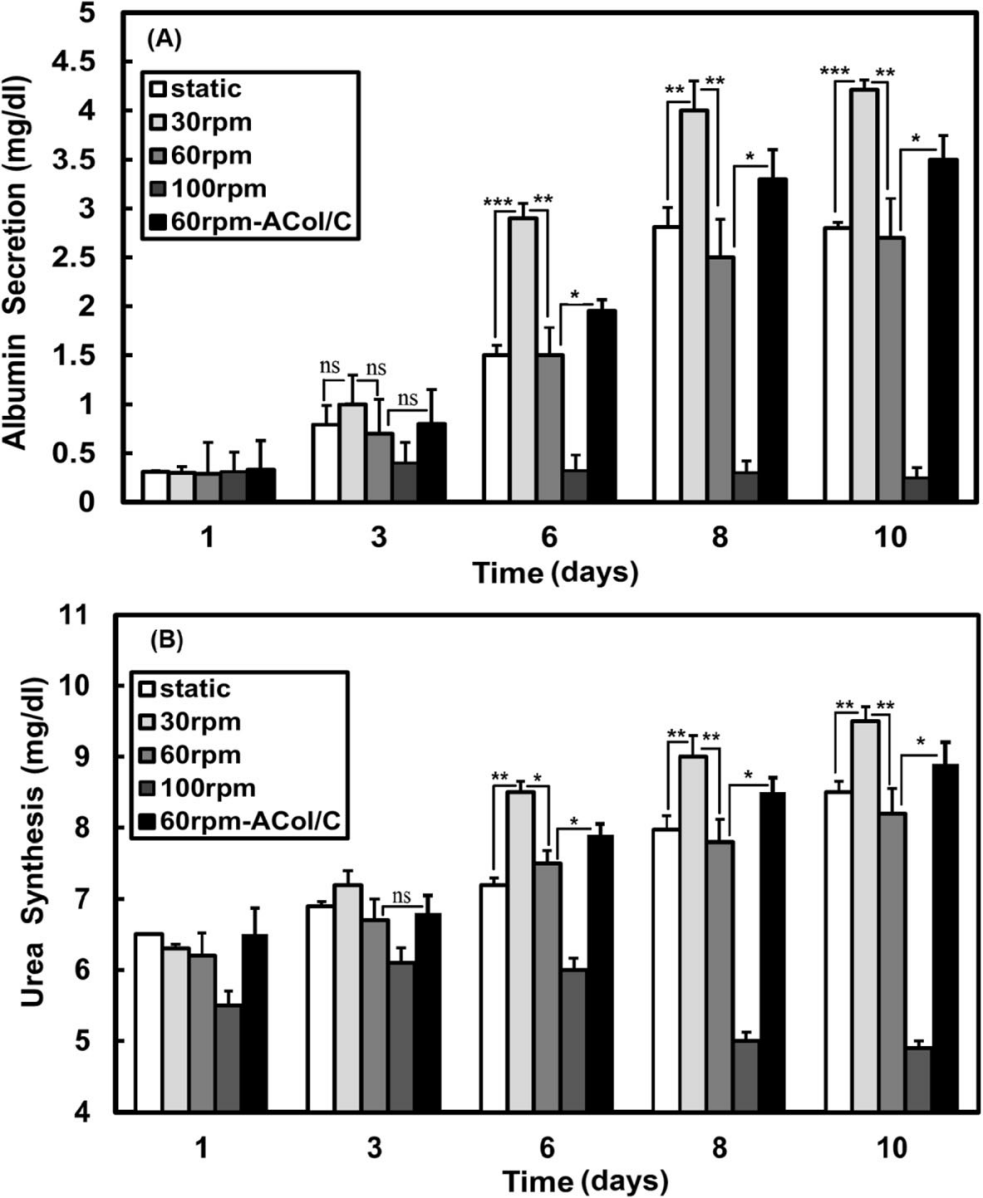
Liver-specific secretions of albumin (**a**) and urea (**b**) for microencapsulated HepG2 cell line after 10 days in the stirred bioreactor

Albumin and urea secretion are of the important indicators for evaluating hepatocyte health and function. Albumin is used as a liver function to indicate liver pathology and it is synthesized almost entirely by the liver [24, 36]. To survey the effect of agitation rate on the liver-specific functions of the microencapsulated HepG2 cells, albumin secretion and urea synthesis were quantified at different mixing rates. Figure 4a represents the amount of albumin secreted into the medium on the days of analysis.

After 3 days, the albumin secretion of microencapsulated HepG2 cells cultured at 30 rpm agitation rate was significantly greater than all conditions (*p* < 0.05), whereas on day 3, this difference was not significant. On day 3, the difference in the albumin secretion of the cell-laden microcapsules between 30 and 100 rpm speeds was not statistically significant (*p* > 0.05), demonstrating the microencapsulated cells maintained their hepatic functions until day 3 when they were cultured at 100 rpm speed in the bioreactor. On day 10, the albumin secretion at 30 rpm culture was significantly higher than the control group and 60 rpm cultures (*p* < 0.01). Accordingly, it may be concluded that the microencapsulated hepatic cells at 30 rpm culture exhibit high viability as well as active liver specific functions. The amount of albumin secretion for the ACol/C microcapsules was significantly higher than the AC microcapsules at 60 rpm agitation rate after day 3 (*p* < 0.05). Urea synthesis is another important liver-specific function of HepG2 cells. The urea synthesis profile of the microencapsulated cells was observed similar to that of the albumin secretion. As it can be seen in Fig. 4b, the function of urea synthesis by the cell-laden AC microcapsules at 30 rpm speed was greater than the static and the dynamic cultures of 60 and 100 rpm on day 6 and thereafter (*p* < 0.05). During the culture period, the urea synthesis of the AC-microencapsulated cells at 60 rpm rate showed no significant difference in comparison with the static culture. During the culture period, the urea synthesis for ACol/C microcapsules was higher than that of AC ones at 60 rpm rate, so that the difference was significant (*p* < 0.05) from the 6th day. On day 10, the urea synthesis was significantly higher at 30 rpm speed compared to ACol/C microcapsules (*p* < 0.05). The liver-specific function results were observed in accordance with the obtained results of the microencapsulated cell proliferation, indicating the importance of microcapsule core composition and mixing rate in the dynamic cultures of microencapsulated hepatic cells.

### Gene expression of microencapsulated HepG2 cells

**Fig. 5:**
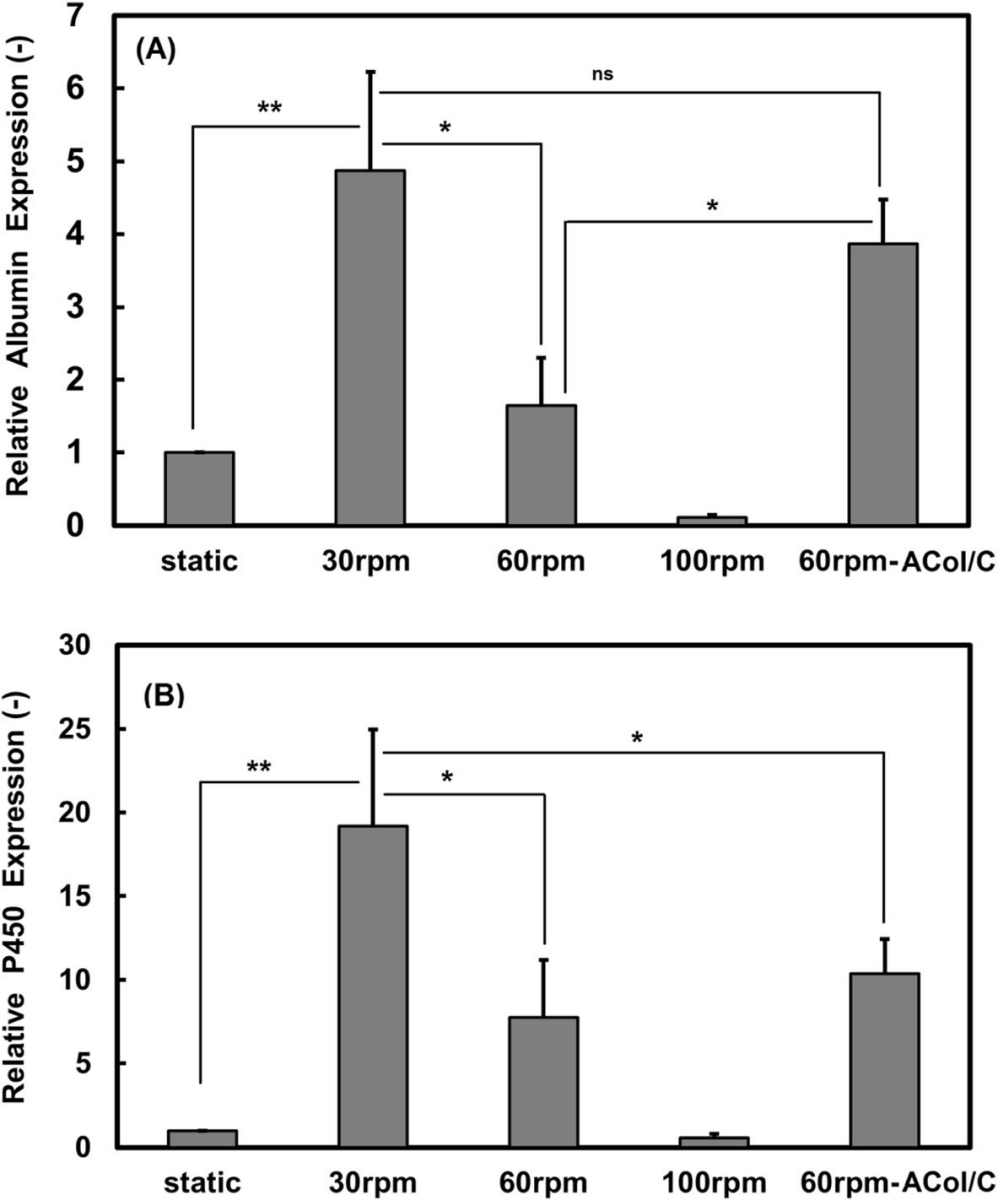
Real time PCR analysis of albumin and P450 expression in microencapsulated HepG2 cells inside AC and ACol/C microcapsules after 10 days dynamic culture

Gene expressions of albumin and cytochrome P450 were determined after 10 days dynamic culture of the microencapsulated hepatocyte model cells. Among the groups, the highest expression of both genes was found at 30 rpm agitation speed culture. Based on the data obtained from the real-time PCR analysis, 4.9-fold increase was observed for the expression of albumin at 30 rpm speed which was statistically significant as compared to other dynamic conditions (Fig. 5a; *p* < 0.05). The ACol/C microcapsules at 60 rpm speed could significantly induce the expression of albumin 46% higher than the AC microcapsules at the same mixing rate (*p* < 0.05). As it is clear from gene expression panel, there is significant difference in the p450 gene expression of microencapsulated hepatic cells cultured at 30 rpm speed with all the other groups (*p* < 0.05). The expression of p450 gene in the AC and ACol/C microcapsules cultured at 60 rpm were 7.7- and 10.4-fold, respectively, as compared to the static culture. In contrast to the expression of albumin, the P450 gene expression in the AC and ACol/C microcapsules cultured at 60 rpm speed was not significant (Fig. 5b).

## Discussion

Maintaining liver-specific function in vitro has been attempted in many researches. Formation of hepatocyte spheroids in 3D cultures has been shown a promising approach for proliferation, and enhancing liver-specific functions [37]. Based on the our previous study, alginate-chitosan core-shell hydrogel microcaspule showed a potential 3D paltform for hepatic cell proliferation in static culture and collagen could improve the 3D microenvironment for use in hepatic tissue engineering [18]. Due to the limitations of static culture, dynamic system can provide better condition for hepatic cell expansion while maintaining liver specific-functions [24, 25].

In this work, we examined alginate-chitosan core-shell hydrogel microcaspule for hepatic cell proliferation in a small scale stirred bioreactor and investigated the influence of shear force and collagen presence in the microcapsule core composition on the hepatic cell proliferation and function. Although shear stress is an important biomechanical parameter influencing hepatocyte function, its impacts on microencapsulated hepatic cells in bioreactors are not well studied.

There have been few reports on the influence of fluidic shear forces on stem cells and the mechanism behind this. Several studies have shown that the bioreactor culture system affects the influence of shear rate on stem cells [27]. Sikavitsas, Bancroft [38] investigated the effect of cell culture condition of 3D PLGA polymers with MSCs in spinner flask and rotating wall vessel bioreactor. The spinner flask culture demonstrated a 60% enhanced proliferation at the end of the first week when compared to static culture, while the culture condition in the rotary bioreactor was not favorable for MSCs cells.

The aforementioned results showed that at high agitation rate (100 rpm) in the stirred bioreactor, encapsulation matrix could not protect the hepatic cells from shear force damages. The results of cell behavior in the ACol/C microcapsules revealed that the matrix composition can affect cellular behavior at the same shear rate. For example, at the agitation rate of 60 rpm, the amounts of albumin secretion on days 6 and 8 for the microcapsules containing collagen were about 25 and 30% higher than that of alginate alone, respectively.

Studies show cell aggregate characteristics are quite dependent upon hydrodynamics. When agitation rate increases; a larger number of smaller and denser aggregates are formed [39, 40]. Agitation is required to keep the microcapsules uniformly suspended and to assure a homogeneous environment for cell growth in dynamic cultures. Stirred bioreactors, in different geometries and scales, operate on the same principal mainly based on the required energy for agitation of medium provided by a rotating stirrer [41]. As a result of the impeller movement, primary eddies are formed and the flow certainly is not laminar. The flow in stirred bioreactors for stem cell culture can be reasonably considered as moderately turbulent [29, 41–46].

The mixing within bioreactors which can generate shear stresses leading to cell damage is influenced by the rotation speed, vessel design and time of culture. The primary mechanisms of cell damage in cell-laden micro-carrier cultures within bioreactor appear to result from direct interaction between micro-carrier and turbulent eddies, collisions between micro-carrier and collisions between impeller and micro-carrier [42, 47]. Analysis of impact of stress generated by agitation on cell culture is usually based on Kolmogorov’s theory of isotropic turbulence [41, 42, 45]. This theory has been successfully applied to bacteria, yeast, and animal cells [41, 48]. The smallest size of micro-eddies in a turbulent flow is called a Kolmogorov scale, which may be evaluated by the following equation [49]:

5 $$ {\lambda}_k={\left(\frac{\vartheta^3}{\varepsilon}\right)}^{\frac{1}{4}} $$

where *ε* is the dissipation rate of kinetic energy and *υ* is the kinematic viscosity.

Some reports show that damage of cells increases with increasing micro-carrier size, agitation rate and amount of micro-carrier loading, and damage became significant when the micro-eddy is about two-thirds of the micro-carrier size, or smaller [33, 50, 51].

The calculations for hydrodynamic parameters are summarized in Table 2 by considering microcapsule as a micro-carrier. The estimated micro-eddy values (*λ*_*K*_), at 30 and 100 rpm agitation rates were 345 and 146 μm, respectively, which were the maximum and minimum eddy sizes in this study. At 100 rpm rate, the size of eddies was approximately 70% lower than the Kolmogorov’s scale threshold (two-thirds of microcapsules diameter). Microcapsules, therefore, will experience shear force on their surface. The resulting force may kill or damage cells in microcapsules especially the cells that are near the microcapsule surface. In addition to eddy shear, other damage phenomena are inter-particle collisions; collisions with walls, other stationary surfaces and the impeller [41, 47]. At high agitation rate, elastic microcapsules have high velocity collision with impeller and other microcapsules. Collisions of the microcapsules against the impeller or other bioreactor internals have a similar effect as microcapsule-microcapsule collisions. Since the surface of impeller and bioreactor are not elastic; the kinetic energy of the collision is much higher. Although the exact mechanism of fluidic shear force on microencapsulated cells inside the elastic beads is not completely clear, hydrogel bead size and components as well as cell concentration can modulate the transmittance of mechanical forces to the microencapsulated cells in dynamic culture [52]. In liquid core microcapsules, however, encapsulated cells can freely move inside the microcapsules and proliferate in a 3D structure [53].

**Table 2:** The calculated values of hydrodynamic parameters at different speeds

Impeller speed (rpm)	Reynolds number	Specific energy dissipation rate(W kg^−1^) × 10^−4^	Maximum shear stress τ_max_ (pa) × 10^−2^	Microscale of turbulence λ_K_ (μm)	Integrated shear factor ISF(s^−1^)
30	670	0.544	3.72	345.23	4.71
60	1340	3.90	10.2	210.43	8.72
100	2270	16.5	20.5	146	14.7

In micro-carrier culture, Chisti [47] defined a severity of collision to account for collisions between micro-carriers, and interactions between micro-carriers and internal of a bioreactor in stirred vessels. These possible causes of cell damage can be mentioned in suspended microcapsules culture in stirred bioreactors. Two collision severities were defined as a turbulent collision severity (TCS) for turbulence-associated particle-to-particle impacts, and an impeller collision severity (ICS) for particle-to-impeller collisions. These indices are proportional with *N*^*4.5*^ and *N*^*4*^, respectively. Studies show when the concentration of solids in suspension exceeds 20% (by volume), the dominant mechanism of cell damage is particle-particle interactions [54]. In our study, the loading of microcapsules in the bioreactor system was 5%. At low agitation rates, therefore, TCS index was minimum and particle-particle interaction was not effective in the cellular damage, while at high agitation rates (100 rpm), microcapsule-microcapsule collision can be a reason for the cellular damage.

Croughan, Hamel [33] reported 18 s^− 1^ as damaging threshold ISF value according to Eq. (4) for micro-carrier-supported fibroblasts. In the present study, the bioreactor dimensions were designed in such a way to have the ISF value as possible as minimum. As it can be seen, the maximum value of ISF for the bioreactor culture was under the threshold value reported for fibroblasts cells (Table 2).

The results of the present study showed when the HepG2cells were cultured at relatively low agitation speed (30 rpm), they achieved higher expansion folds and better liver-specific functionality. Although high rotational speed increases the gas-liquid mass transfer in the bioreactor, the choice of higher rotational speed has a major impact on the magnitude of mechanical constraints.

Influence of substrate mechanics on the response of HepG2 cells to fluid shear stress is also noteworthy. The results revealed the effect of combination of matrix stiffness and shear rate on behavior of hepatocytes. Addition of collagen to the microcapsule core composition could improve matrix stiffness and consequently the cell-laden microcapsules showed better the cell proliferation and functionality at 60 rpm in comparison with the cell-laden microcapsules without collagen.

It is interesting to know, Galie, Van Oosten [55] used a novel microfluidic device to investigate the combination of substrate stiffness and shear stress on the inflammatory response of endothelial cells. To further explore this effect, they plated the cells on three levels of substrate stiffness (100 Pa, 2.5 kPa, and 10 kPa) and then the cells were exposed to various shear forces. Interestingly, they reported that the cells responded differently to shear stress increase on soft substrate while the cells showed no detectable changes on stiff substrate by the same increase in shear stress. According to our previous study [18] and the result of mechanical properties in the present work (Fig. 3b), ACol hydrogel is stiffer than alginate alone and this difference in mechanical properties can affect the cell response to shear rate. In addition to the mentioned reason, collagen as the most abundant component in liver tissue has adhesion motifs such as RGD (Arg-Gly-Asp) that increases interaction of cells with ECM and regulates functional behavior of hepatocytes. The results of present study suggest that substrate mechanics can modulate the effects of shear stress in the stirred bioreactor.

## Conclusions

In this study we studied the response of HepG2 cells cultured in alginate-chitosan core-shell microcapsule to shear force at different agitation rates. Our study demonstrated that albumin production, one of the specific hepatocyte functions, was enhanced at low agitation rate in comparison with that in the static culture and other agitation rates. Besides, it was found that the microcapsule core composition could modulate shear stress influence on the microencapsulated cell behavior in the dynamic culture. In conclusion, the present study demonstrated that culture of hepatic cells in alginate-chitosan core-shell microcapsule under controlled shear condition in the stirred bioreactor can increase proliferation and expression of liver-specific functions and can be a promising strategy for mass production of hepatocytes cells although further experiments are needed.

## Data Availability

The datasets used and/or analyzed during the current study are available from the corresponding author on reasonable request.

## Abbreviations

A: alginate
C: chitosan
Col: collagen
AC: alginate/chitosan
ACol/C: alginate+collagen/chitosan
ECM: Extracellular matrix
HepG2 cells: Hepatoma G2 cells
H-DMEM: High glucose-Dulbecco’s Modified Eagle’s Medium
MTT: 3-[4,5-dimethyl-2-thia-zolyl]-2, 5-diphenyl-2H-tetrazolium bromide
